# Development of an In Situ Photo-Crosslinking Antimicrobial Collagen Hydrogel for the Treatment of Infected Wounds

**DOI:** 10.3390/polym15244701

**Published:** 2023-12-13

**Authors:** Song-Yi Wu, Wei-Bor Tsai

**Affiliations:** 1 Department of Chemical Engineering & Program of Green Materials and Precision Devices, National Taiwan University, No. 1, Sec. 4, Roosevelt Rd., Taipei 106, Taiwan; zaser789@gmail.com; 2 Guangdong Victory Biotech Co., Ltd., 4F., A11, Guangdong New Light Source Industrial Park, Luocun, Shishan Town, Nanhai District, Foshan 528226, China; 3 Guangxi Shenguan Collagen Biological Group Company Limited, No. 39 Xijiang 4th Rd., Wuzhou 543099, China

**Keywords:** antimicrobial peptide, antibiotics, hydrogel, infected wound, wound healing

## Abstract

Antimicrobial hydrogels have received considerable attention in the treatment of bacteria-infected wounds. Herein, we develop a neutral, soluble collagen via modification with maleic anhydride, serving as a hydrogel precursor. Maleic anhydride-modified collagen (ColME) could form a gel after exposure to UV light and be loaded with the antimicrobial agents, nisin and levofloxacin, to acquire antimicrobial ability. The ColME hydrogel containing nisin and levofloxacin had good cytocompatibility and effectively killed pathogenic bacterial strains, such as *Escherichia coli* and *Staphylococcus aureus*. The antimicrobial ColME hydrogels effectively supported the healing of a full-thickness skin wound infected with *S. aureus* in a mouse model. Our results demonstrate the potential of antimicrobial hydrogels as effective wound dressings via in situ photogelation for the healing of infected wounds.

## 1. Introduction

Wound infection is one of the main obstacles to a typical healing process [1]. Harmful microorganisms such as bacteria, invade and multiply within a wound, leading to increased pain, redness, swelling, delay in the healing process, and potentially other serious complications. Chronic wounds are particularly vulnerable to infection and can be difficult to treat. Healthcare providers must ensure the proper diagnosis and management of wound infections in order to effectively prevent additional complications. [2,3].

Wound dressings are required for the treatment of skin wounds to promote healing, prevent infection, and protect the wound from external hazards such as dirt and bacteria [4]. Conventional wound dressings, such as gauze, cotton wool, and natural or synthetic bandages, may not provide optimal wound care due to inadequate moisture control, limited absorbency, and inadequate protection against external factors. Furthermore, such wound dressings often adhere to wound beds and then cause discomfort, and potential pain or trauma when removed [5,6]. In this respect, hydrogel dressings outperform conventional wound dressings due to their unique properties, including high water content, hydrophilicity, softness, flexibility, biodegradability, and biocompatibility. The high water content of the hydrogel creates a moist wound environment which prevents wound dehydration, reduces the risk of scab formation, and enables the natural healing process to occur. Additionally, the excellent fluid retention ability of hydrogel dressings allows them to absorb excess exudate while maintaining an optimal moisture balance at the wound site. Hydrogels are easy to apply to and remove from a wound, minimizing the risk of trauma to the wound during dressing changes. Hydrogels are also suitable for all stages of the healing process, from hemostasis to inflammation, proliferation, and remodeling. These advantageous characteristics establish hydrogel dressings as a superior choice for effective wound management [6,7,8].

In the traditional way, hydrogels are prefabricated and placed on target wounds in patients, which may not fully cover uneven or irregular wound surfaces, resulting in ineffective wound healing and low cell survival for tissue remodeling [9]. The problem could be overcome by applying hydrogel precursors that form hydrogel in situ. The precursor solution could fill uneven or irregular wound beds and facilitate tissue regeneration [10]. Among in situ cross-linking methods, photoinitiated cross-linking is a promising strategy due to good spatiotemporal control and rapid induction under mild conditions [11]. To this end, natural polymers, such as gelatin and hyaluronic acid, are conjugated with methacrylate for the fabrication of photo-initiated hydrogels [12,13,14]. On the contrary, collagen, a vital extracellular matrix protein in many biological activities, such as biomechanical support, hemostasis, and cell affinity [15], is seldom used as a photogelation precursor, although collagen has been widely applied to wound dressings on the market [16]. The main reason is the low solubility of methacrylated collagen in a neutral or physiological solution. Therefore, methacrylated collagen must first dissolve in an acidic solution and then be adjusted to neutral pH before application [17,18,19,20]. The inconvenience could be overcome by modifying collagen with unsaturated cyclic anhydrides, such as maleic anhydride. The reaction of maleic anhydrides with amines in collagen to form amide bonds not only brings methacrylate groups to collagen but also replaces amines with carboxylates [21]. Consequently, the isoelectric point of collagen changes from neutral pH to a lower pH [22,23]. In the previous study, collagen modified with maleic anhydride still needs to be first dissolved in acid buffers due to the low substitution ratio (~10%) [21]. The introduction of carboxyl groups improved the anionic characteristics of collagen, thereby lowering its isoelectric point and allowing solubility at the physiological pH level [20,24]. A further increase in substitution ratios could make soluble methacrylated collagen dissolve at neutral pH as a precursor for photoinitiated gelation.

**Scheme 1:**
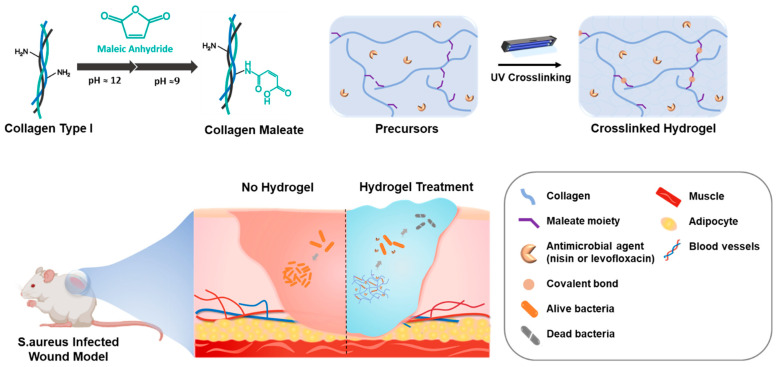
Schematic illustration highlighting the process of ColME synthesis, fabrication of antimicrobial hydrogels, and its application for the effective healing of infected wounds.

The main objective of this study was to develop a collagen-based hydrogel precursor that could encapsulate antimicrobial agents and undergo gelation in situ via photostimulation for the treatment of infected wounds (Scheme 1). Two antimicrobial agents, nisin and levofloxacin, were used for encapsulation. Nisin, an antimicrobial peptide synthesized by *Lactococcus lactis*, has gained recognition as a food additive, with approval from both the US Food and Drug Administration (FDA) and the World Health Organization [25], and exhibits remarkable efficacy against a wide range of Gram-positive bacteria [26]. Positively charged nisin exhibits an affinity for negatively charged bacteria, initiating cell membrane disruption and consequent bacterial demise [27,28]. Nisin has been shown to effectively reduce bacterial growth and promote wound healing by decreasing the concentration of pro-inflammatory cytokines [29]. Levofloxacin, classified as a fluoroquinolone antibiotic, exhibits broad-spectrum activity against various bacterial pathogens. Levofloxacin is also approved by the FDA for the treatment of several medical conditions and has been used in wound dressings as a therapeutic approach to the treatment of infected wounds, highlighting its potential role in the healing of infected wounds [30,31,32]. 

In this research endeavor, we present the synthesis of an innovative collagen-based hydrogel that has been strategically modified through maleic anhydride functionalization and fortified with antimicrobial agents, with the primary objective of addressing the treatment of infected wounds. The maleic anhydride modification of collagen was meticulously executed, resulting in a significantly heightened degree of substitution. This modification endows the resultant collagen derivative with the remarkable ability to readily dissolve in a neutral buffer solution. Subsequently, the precursor of the modified collagen hydrogel was judiciously combined with either nisin or levofloxacin and subsequently subjected to crosslinking through the utilization of UV irradiation. This innovative hydrogel formulation offers several noteworthy advantages, including the elimination of the necessity for conventional crosslinking agents, thereby alleviating potential concerns regarding toxicity. Furthermore, it ensures the sustained release of antimicrobial agents, thereby enhancing their therapeutic efficacy. Additionally, this hydrogel system boasts the capability of rapid in situ photoinitiation gelation, thereby facilitating its application to uneven or irregular wound surfaces. The cytocompatibility of the resulting collagen hydrogel encapsulating either levofloxacin or nisin was systematically assessed, and comprehensive investigations were conducted to scrutinize the release kinetics of antimicrobial agents from the hydrogel, as well as their effectiveness against both *S. aureus* and *E. coli*. In order to evaluate the therapeutic potential of this hydrogel in the context of treating infected wounds, experiments were meticulously conducted employing a mouse model featuring full-thickness skin wounds that were intentionally infected with *S. aureus*. Notably, this study introduces an innovative approach, wherein the maleic anhydride-modified collagen hydrogel serves as a highly compatible carrier for antimicrobial agents, effectively fostering the healing of infected wounds.

## 2. Materials and Methods

### 2.1. Materials

Type I bovine atelocollagen was kindly supplied by Guangdong Victory Biotech (Foshan, China). Nisin from Lactococcus lactis (NIS, Cat #N5764), levofloxacin (LEV, Cat #28266), type A gelatin (Gel, Cat #G1890), thiazolyl blue tetrazolium bromide (MTT, Cat #M5655), Luria–Bertani broth (LB, Cat #L3522) and basic fibroblast growth factor (βFGF, Cat #F0291) were purchased from Sigma-Aldrich (St. Louis, MO, USA). Acetic acid (HOAc, Cat #33209) was purchased from Honeywell Fluka (Morris Plains, NJ, USA). Lithium phenyl-2,4,6-trimethylbenzoylphosphinate (LAP, Cat #L0290, a photoinitiator) and N-succinimidyl methacrylate (NSM, Cat #S0812) were purchased from Tokyo Chemical Industry (Tokyo, Japan). Maleic anhydride (MA, Cat #A12178.30) was purchased from Thermo Fisher Scientific (Waltham, MA, USA). LIVE/DEAD cell imaging kit (Cat #R37601) was purchased from Invitrogen (Carlsbad, CA, USA). Bradford reagent (Cat #5000006) was purchased from Bio-Rad (Hercules, CA, USA). Dulbecco’s modified Eagle’s medium (DMEM, Cat #12100-046) was purchased from Gibco (Grand Island, NY, USA). Fetal bovine serum (FBS, Cat #04-001-1A) was purchased from Biological Industries (Kibbutz Beit Haemek, Isreal). Antibiotic–antimycotic solution (Cat #SV3007901) was purchased from Hyclone (Logan, UT, USA). Tegaderm (Cat #90600) was purchased from 3M Health Care (Maplewood, MN, USA). The bacterial strains used in this study were *Staphylococcus aureus* (*S. aureus*, ATCC21351) and *Escherichia coli (E. coli*, ATCC23501). All other chemicals were purchased from Sigma-Aldrich unless otherwise specified.

### 2.2. Synthesis of Collagen Maleate (ColME) and Gelatin Methacrylate (GelMA)

Collagen maleate (ColME) was synthesized following a previous method [21]. Briefly, collagen was dissolved in 0.5 M acetic acid at a concentration of 0.8% (*w*/*v*) and then the pH was adjusted to 11.7. Next, 1 M MA in acetone was gradually added to the collagen solution and the pH was adjusted to 9.0. The mixture was then incubated overnight at 4 °C with continuous gentle stirring. ColME was dialyzed against phosphate buffered saline (PBS) at 4 °C for 3 days with several PBS exchanges. Gelatin methacrylate (GelMA) was prepared following a previous method [33]. Briefly, type A gelatin and NSM were dissolved in PBS and dimethyl sulfoxide, respectively, and then mixed to form a solution of 0.1% (*w*/*v*) gelatin and 0.25% NSM (*w*/*v*). The mixture was allowed to react overnight at room temperature under constant stirring. The resulting product was purified by dialysis with reverse osmosis water for 3 days, followed by lyophilization.

### 2.3. Preparation and Characterization of Hydrogels

A ColME hydrogel precursor solution containing 0.7% (*w*/*v*) ColME and 0.06% (*w*/*v*) LAP was prepared, while a GelMA hydrogel precursor solution containing 3.2% (*w*/*v*) GelMA and 0.06% (*w*/*v*) LAP was prepared. Hydrogel was formed by exposure of the precursor solution to 365 nm UV light for 1 min, which activated LAP to initiate polymerization of ColME hydrogel precursors. For preparation of antimicrobial hydrogels, ColME-NIS was prepared by adding nisin into the precursor solution of ColME hydrogel at concentration of 0.125, 0.25, 0.5 and 1 mg/mL. Similarly, ColME-LEV was prepared by adding levofloxacin to the precursor solution of ColME hydrogel at concentrations of 5, 10, 50, 100 and 1000 μg/mL. GelMA hydrogels loaded with either nisin or levofloxacin were not prepared in this study. The inclusion of GelMA hydrogel was specifically conducted to evaluate the in vivo wound healing efficacy of gelatin in comparison to collagen.

The functionalities of ColME, nisin, levofloxacin, ColME-NIS (1 mg/mL) and ColME-LEV (0.1 mg/mL) were analyzed by Fourier transform infrared (FT-IR) spectra scanning from 4000 cm^−1^ to 500 cm^−1^ at room temperature with 1 cm^−1^ resolution using an FT-IR spectrometer (Spectrum 100, PerkinElmer, Waltham, MA, USA) and attenuated total reflectance infrared spectroscopy (ATR-IR).

### 2.4. In Vitro Studies of the Release of Antibacterial Agents

To determine the release of antibacterial agents from hydrogels, a hydrogel made from 200 μL of a ColME hydrogel precursor solution was placed into 1 mL of PBS and incubated at 37 °C with continuous shaking. At specific time intervals, the PBS was carefully collected and substituted with fresh PBS. The release of nisin (ColME-NIS, 1 mg/mL) and levofloxacin (ColME-LEV, 0.1 mg/mL) was determined according to the absorbance at 595 nm using the Bradford assay and an absorbance of 285 nm, respectively (UV–Visible spectrophotometer, Cary 300, Agilent, Santa Clara, CA, USA). 

### 2.5. In Vitro Studies of Antimicrobial Efficacy

The antimicrobial activity of ColME, ColME-NIS, and ColME-LEV hydrogels against *S. aureus* (Gram positive) and *E. coli* (Gram negative) was tested using an inhibition zone method and a colony counting method. In the inhibition zone method, 500 μL of bacterial suspension (2 × 10^7^ CFU/mL) was spread uniformly on a solid LB agar plate and then covered with a hydrogel sample, followed by incubation at 37 °C for 24 h. The diameter (mm) of the inhibition zone was then measured to determine the antimicrobial efficacy of the hydrogel. In the colony counting method, a hydrogel made from 400 μL of a ColME hydrogel precursor solution was immersed in 2.4 mL of bacterial suspension (10^4^ CFU/mL) at 37 °C for 6 h. The resulting bacterial suspension was subjected to dilution and evenly spread on an LB agar plate, and then incubated at 37 °C overnight. Bacterial colonies were counted on the plates. The antimicrobial activity of a sample was quantified using the following equation,

(C_C_ − C_S_)/C_C_ × 100% (1)

where C_C_ and C_S_ represent CFUs in the control and hydrogel groups, respectively. 

### 2.6. In Vitro Cytocompatibility

The cytocompatibility of the hydrogels was assessed using the MTT assay with the L929 cell line, following the guidelines in ISO 10993-5 [34]. Both the extraction and direct contact methods were employed. In the extraction test, the hydrogels were fully immersed in DMEM supplemented with 10% FBS and 1% (*v*/*v*) antibiotic–antimycotic solution at 37 °C for 24 h, and the extracts were collected. L929 fibroblasts were cultured in 96-well plates at a density of 2 × 10^3^ cells/well with 100 μL of the extract solution. After 24 h of incubation, the culture medium was replaced with MTT solution for a 4 h reaction. The crystals formed were dissolved by dimethyl sulfoxide and quantified by absorbance at 570 nm. Cytotoxicity was evaluated by comparison of the absorbance from the hydrogels with that of the control.

In the direct contact test, L929 fibroblasts were seeded on the hydrogels at a density of 2 × 10^3^ cells/well for 24 or 72 h of incubation, followed by the MTT assay to determine cell viability. Cells were stained with a LIVE/DEAD staining reagent according to the manufacturer’s instructions. The fluorescent images were visualized using an inverted fluorescence microscope (IX 71, Olympus, Tokyo, Japan). 

### 2.7. In Vivo Wound Healing in a Bacterial-Infected Mouse Model

A full-thickness bacteria-infected wound model was established using a mouse model to evaluate the effect of an antimicrobial hydrogel on wound healing. Animal care and experimental procedure were approved by the Institutional Animal Care and Use Committee of the National Taiwan University (Approval No. NTU-111-EL-00023). Balb/c male mice (aged 8 weeks) were anesthetized by inhalation of isoflurane. After the removal of the dorsal hair, a full-thickness skin wound (~0.8 cm in diameter) was created on the depilated back skin. Mice were randomly assigned to five groups (3 mice/group): Control (no hydrogel), ColME-NIS, ColME-LEV, GelMA and ColME. A volume of 20 μL of *S. aureus* suspension (~2 × 10^7^ CFU/mL) was inoculated into each wound, followed by the application of a hydrogel precursor solution and gel formation by exposure to 365 nm UV light for 1 min, followed by covering the wound with a Tegaderm film. The hydrogels were replaced with new ones on Days 3, 7 and 11. The wounds were photographed on days 0, 3, 7, 11 and 14. The acquired images were analyzed using ImageJ software 1.8.0 to determine the size of the wounds. The degree of wound closure was calculated using the following equation:

Wound closure (%) = (S_0_—S_n_)/S_0_ × 100%,

where S_0_ and S_n_ represented the wound areas on day 0 and day 3, 7, 11 or 14, respectively.

To evaluate the antimicrobial efficacy of hydrogels in vivo, wound exudates were collected using sterile cotton swabs on day 3 and 7. The swab was gently rotated at the wound site to absorb exudate, ensuring that it was in contact with only the exudate and avoided scraping against the wound bed or edges. The process was carefully carried out to prevent any removal or disruption of the biofilm formation. The collected samples were immersed in LB broth and then inoculated onto agar plates with appropriate dilutions, followed by overnight incubation at 37 °C. The bacterial colonies were counted on the plates. 

On day 14, mice were sacrificed by CO_2_ inhalation. Skin tissue samples were harvested and fixed with a 4% paraformaldehyde solution for the preparation of histological slides. The slides were subjected to H&E staining, as well as Masson’s trichrome staining. Images of stained samples were captured with an optical microscope (Nikon Eclipse TS 100, Nikon, Tokyo, Japan).

### 2.8. Statistical Analysis

All data were presented as mean value ± standard deviations (SD). Statistical difference was analyzed using the Student’s *t* test in GraphPad Prism 8.0. *p* < 0.05 was considered statistically significant.

## 3. Results and Discussion

### 3.1. Preparation and Characterization of Hydrogels

Modification of bovine type I collagen with maleic anhydride reached a substitution of more than 90%. At this substitution ratio, ColME could be dissolved in neutral buffers. FT-IR spectra were used to analyze the functional groups present in the hydrogels (Figure S1). In the ColME spectra, characteristic peaks corresponding to collagen were identified, including the amide A peak at 3370 cm^−1^ for the N–H stretching vibration, amide I peak at 1654 cm^−1^ for the C=O stretching vibration and amide II peak at 1544 cm^−1^ for N–H bending and C–N stretching. The presence of all of the aforementioned characteristic peaks found in the ColME spectra confirmed the successful preservation of the collagen structure after modification. ColME formed a transparent hydrogel in the presence of LAP Hydrogel by exposure to 365 nm UV light for 1 min (Figure S1 in Supporting Information). To confirm the encapsulation of antimicrobial agents, the spectra of nisin, levofloxacin, ColME-NIS, and ColME-LEV were examined (Figure S2 in Supporting Information). Prominent peaks corresponding to both nisin and levofloxacin were identified, including the peak of amide I at 1654 cm^−1^, the carbonyl C=O peak at 1724 cm^−1^, and the peak of aromatic CH at 2935 cm^−1^ [35,36]. These results showed the encapsulation of the antimicrobial agents in the ColME hydrogel.

### 3.2. In Vitro Release of Antimicrobial Agents and Their Antimicrobial Efficacy

**Figure 1:**
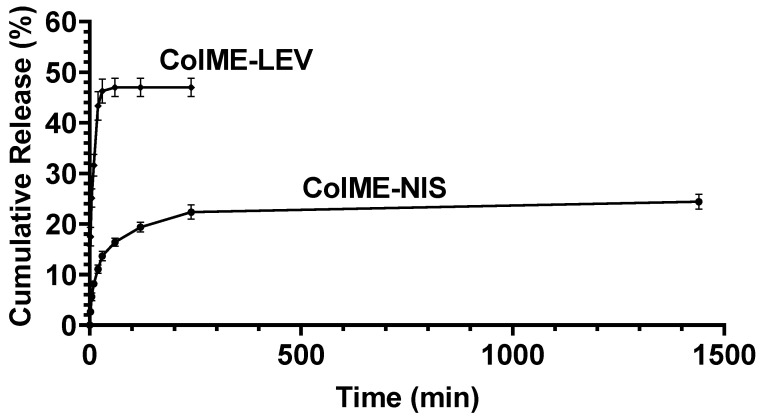
The release profiles of nisin (ColME-NIS, 1 mg/mL) and levofloxacin (ColME-LEV, 0.1 mg/mL) from ColME hydrogel. *n* = 6, error bar = standard deviation.

The release profiles of levofloxacin and nisin from ColME hydrogels are illustrated in Figure 1. The release of small antibiotic, levofloxacin, was very fast, ~50% in 6 min, while the release of the antibacterial peptide, nisin, was much slower, ~25% in 2 days. 

**Figure 2:**
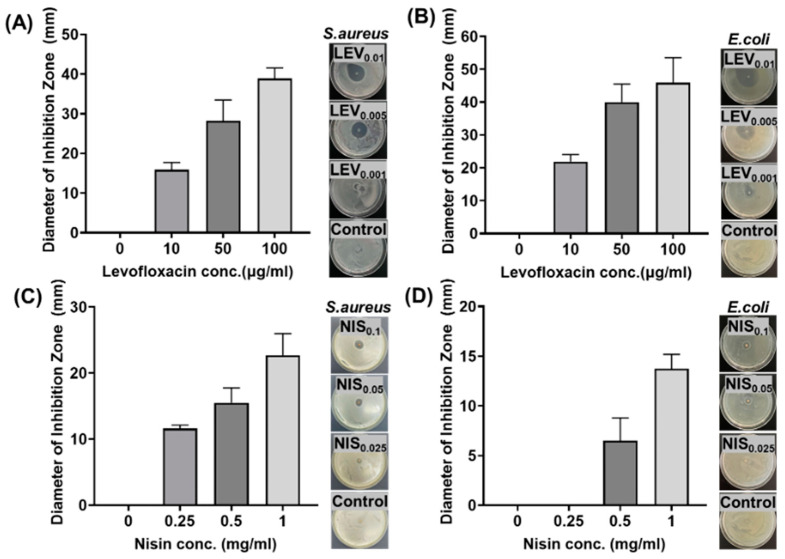
The inhibition zones of (**A**) ColME-LEV against *S. aureus* (**B**) ColME-LEV against *E. coli* (**C**) ColME-NIS against *S. aureus* and (**D**) ColME-NIS against *E. coli*. *n* = 3, error bar = standard deviation.

The antimicrobial efficacy of ColME-LEV and ColME-NIS hydrogels against *S. aureus* (Gram-positive) and *E. coli* (Gram-negative) was assessed through both the colony counting and inhibition zone methods. LEV was encapsulated in the hydrogel at different concentrations, 10, 50 and 100 μg/mL. The diameter of the inhibition zones against *S. aureus* increased with LEV concentrations, from 15.9 ± 1.8, 28.2 ± 5.3 to 38.8 ± 2.7 mm for 10, 50 and 100 μg/mL, respectively (Figure 2A). Similarly, the diameters of the inhibition zones against *E. coli* were 21.9 ± 2.2, 40.0 ± 5.5 and 45.9 ± 7.6 mm for 10, 50 and 100 μg/mL, respectively (Figure 2B). On the other hand, NIS was encapsulated in the hydrogel at different concentrations, 0.25, 0.5 and 1 mg/mL. The diameters of the inhibition zones against *S. aureus* were 11.6 ± 0.5, 15.5 ± 2.2 to 22.7 ± 3.3 mm (Figure 2C), while those were 0, 6.5 ± 2.3 and 13.8 ± 1.4 mm against *E. coli* (Figure 2D) for 0.25, 0.5 and 1 mg NIS/mL, respectively.

**Figure 3:**
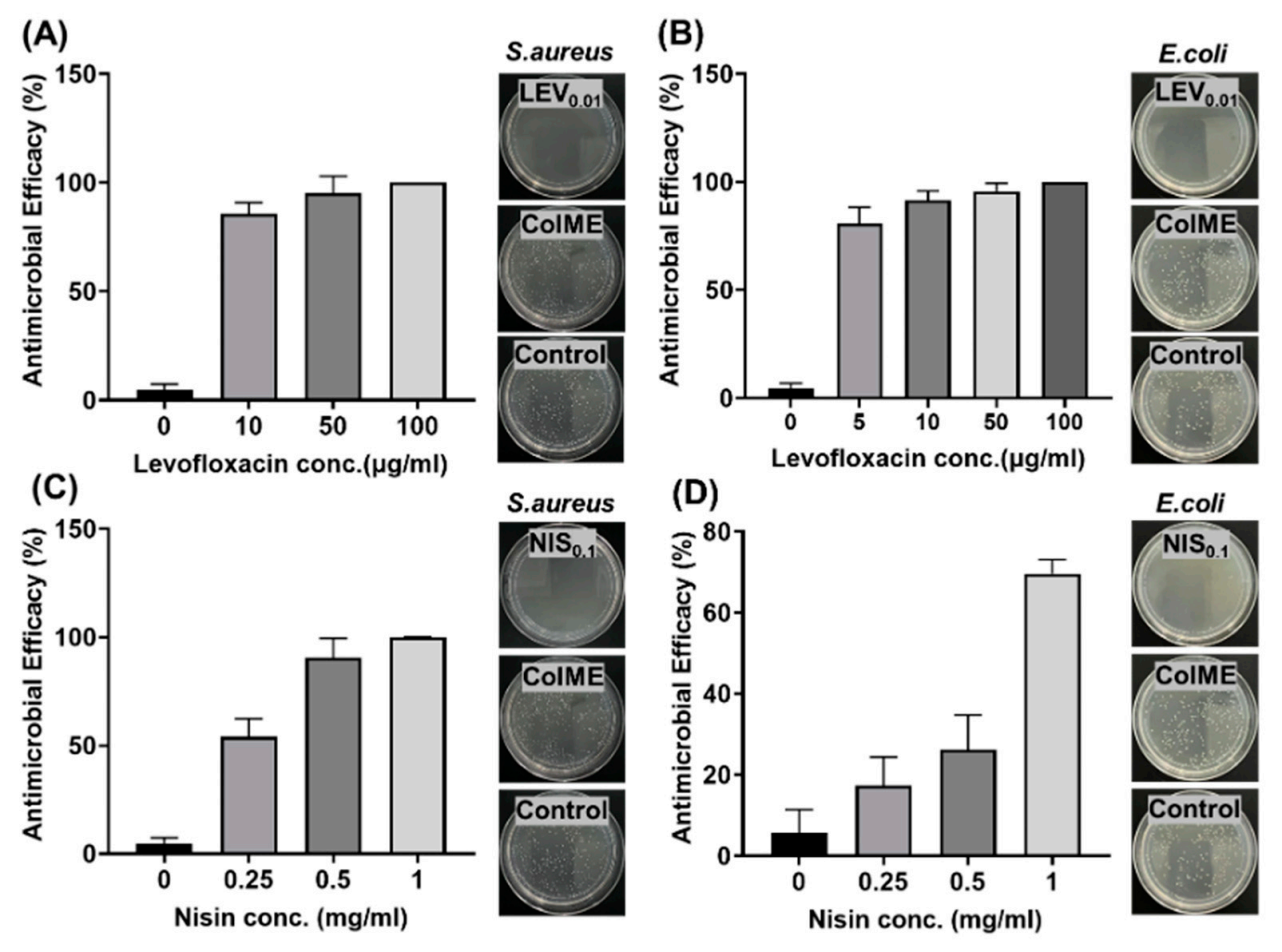
The colony counting tests of (**A**) ColME-LEV against *S. aureus* (**B**) ColME-LEV against *E. coli* (**C**) ColME-NIS against *S. aureus* and (**D**) ColME-NIS against *E. coli*. *n* = 3, error bar = standard deviation.

In the colony counting test, no colonies were found in 100 μg/mL LEV hydrogel against both *S. aureus* and *E. coli* (Figure 3A,B). On the other hand, 10 and 50 μg/mL LEV could not kill *S. aureus* totally (85.7 ± 5.0% and 95.15 ± 7.9%) and *E. coli* (91.6 ± 4.3% and 95.6 ± 3.8%), respectively. In the case of ColME-NIS hydrogels, 1 mg/mL of NIS hydrogel killed all *S. aureus*, but not *E. coli* (Figure 3C,D). Similarly, 0.5 mg/mL NIS hydrogel killed 90.5 ± 9.0% *S. aureus*, or 26.2 ± 8.5% *E. coli*. The results indicate that nisin has a higher antimicrobial efficacy against *S. aureus*. than against *E. coli*.

### 3.3. In Vitro Cytocompatibility Evaluation

**Figure 4:**
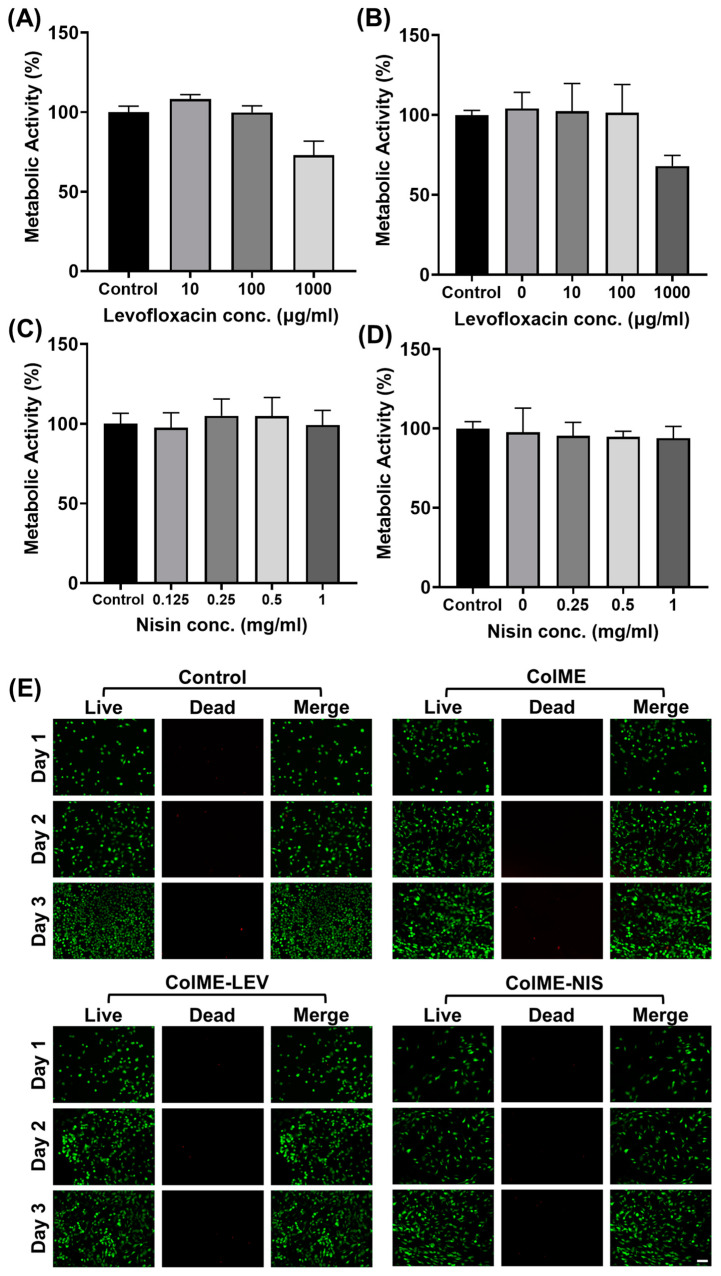
In vitro cytocompatibility tests on hydrogels using the MTT assay. (**A**,**B**) ColME-LEV hydrogels; (**C**,**D**) ColME-NIS hydrogels; (**A**,**C**) the extract method; (**B**,**D**) the direct contact method. *n* = 3, error bars = standard deviation. (**E**) Representative fluorescence images of L929 cells cultured on ColME, ColME-LEV (100 μg/mL), ColME-NIS (1 mg/mL) hydrogels and TCPS control after incubation for 1, 2 and 3 days. (scale bar = 100 μm).

The cytocompatibility of a hydrogel is crucial when applied to wound treatment. The cytotoxicity of ColME hydrogels containing levofloxacin at 10 and 100 μg/mL was negligible using either the extract method (Figure 4A) or the direct contact method (Figure 4B), while 1000 μg/mL of levofloxacin reduced the metabolic activities of L929 cells to ~70%. On the other hand, nisin did not show any cytotoxicity at the concentrations up to 1 mg/mL (Figure 4C,D). L929 cells were also cultured on ColME hydrogels encapsulated with 100 μg/mL of levofloxacin or 1 mg/mL of nisin for 3 days. During the 3-day culture, most cells remained alive (Figure 4E, green fluorescence) and the cell population increased over time. Our results indicate that ColME hydrogel encapsulated with levofloxacin or nisin had good cytocompatibility. 

### 3.4. In Vivo Study on the Healing of Infected Wounds

Before mouse experiments with infection, the efficacy of ColME hydrogel in wound healing was tested. Our results showed that ColME hydrogel greatly facilitated wound healing without bacterial infection (Figure S3 in Supporting Information). Based on the bactericidal efficacy and cytotoxicity of levofloxacin and nisin, ColME hydrogels encapsulated with 100 μg/mL levofloxacin or 1 mg/mL of nisin were then applied to investigate their efficacy in healing infected wounds according to the results of antimicrobial efficacy and cytocompatibility. We previously described in this study that full-thickness skin defects were created on the dorsal side of mice and infected with *S. aureus*, a major pathogen associated with skin wound infections. 

**Figure 5:**
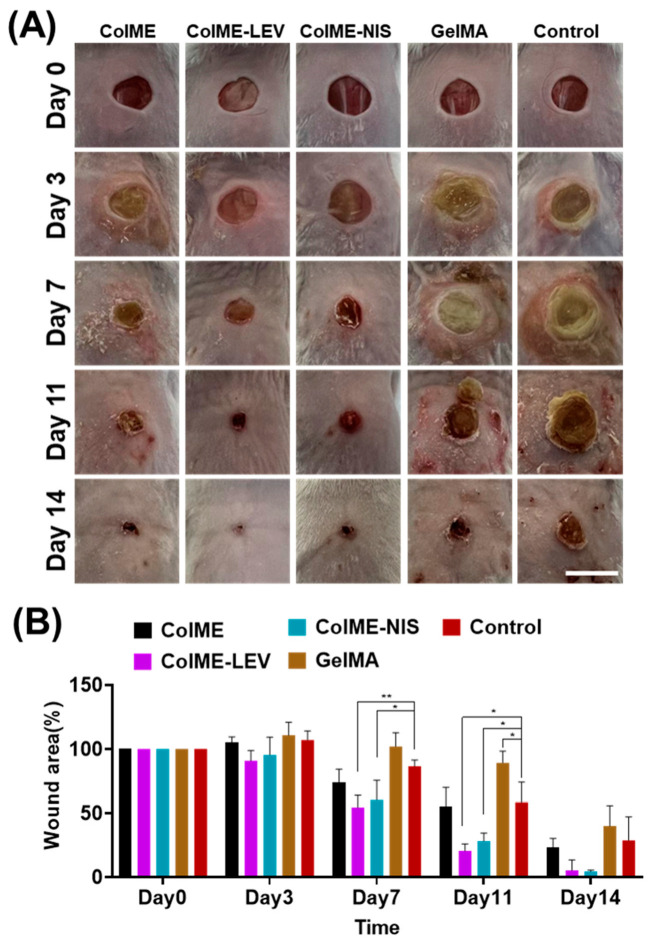
In vivo infected wound healing study. (**A**) Representative images of *S. aureus*-infected wounds with different treatments (ColME-NIS, ColME-LEV, GelMA and ColME hydrogels, and Control) on day 0, 3, 7, 11, and 14 (scale bar = 1 cm). (**B**) The percentage of the wound areas for each group on different days. *n* = 3, * *p* < 0.05, and ** *p* < 0.01.

The wound healing processes in mice were observed and photographed (Figure 5A). The development of biofilm was found in the control group that was not treated with antimicrobial agents, as well as those treated with ColME hydrogel or GelMA hydrogel, in the first week after surgery. Biofilm formation can lead to a delay in the wound healing process [37]. In contrast, incorporation of levofloxacin or nisin inhibited the formation of biofilm, demonstrating an effective antimicrobial activity. The prevention of bacterial infection further facilitated wound closure. In the ColME-LEV and ColME-NIS groups, wound healing progressed noticeably faster than in the other groups, with near-complete recovery observed after 14 days. The wound sizes decreased to 20.5 ± 5.4% in ColME-LEV and 28.2 ± 6.2% in ColME-NIS after 11 days, while the wounds in the other three groups still remained at more than 50% (Figure 5B). After 14 days, ColME-NIS and ColME-LEV achieved almost complete wound healing, while 23.4 ± 7.0%, 40.0 ± 15.9% and 28.7 ± 18.4% wound area remained in ColME, GelMA and Control, respectively. The results demonstrated that the inclusion of antimicrobial agents in ColME hydrogels facilitated the healing of the *S. aureus*-infected wound. One thing to note is that although bacterial infection affected the healing ability of ColME hydrogel without antimicrobial agents, it still had better healing ability compared to GelMA hydrogel in the presence of bacteria. Mice treated with ColME hydrogels exhibited significantly improved wound healing compared to those treated with GelMA hydrogel on day 7 and day 11 (*p* < 0.05), indicating that ColME hydrogel is superior to GelMA hydrogel in facilitating wound healing. 

**Figure 6:**
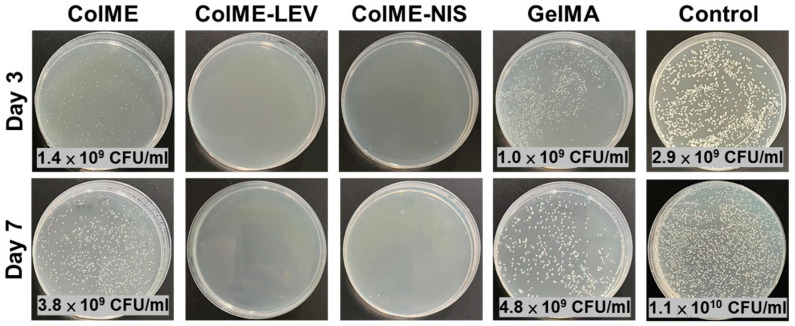
Representative images of bacterial colonies from the infected wounds on day 3 and day 7.

We further investigated the bacteria that survived on wounds on day 3 and day 7 (Figure 6). No bacteria were found in the wounds covered with ColME hydrogels containing antimicrobial agents (ColME-LEV and ColME-NIS), while the groups without antimicrobial agents still contained significant amounts of bacteria. The results demonstrated that the ColME hydrogel encapsulated with nisin or levofloxacin had good antibacterial efficacy and facilitated the healing of infected wounds.

**Figure 7:**
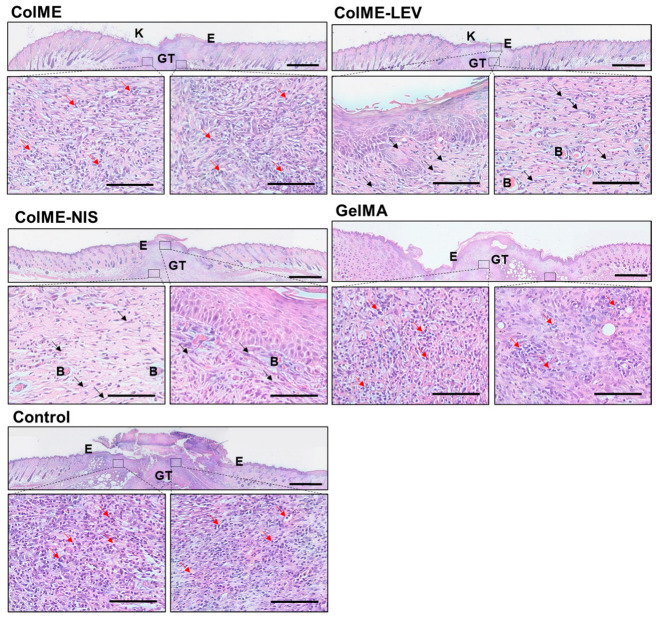
Representative H&E staining images on day 14. Scale bars in the low-magnification images, 1 mm; scale bars in the high-magnification images, 100 μm. Red arrow: inflammatory cells; Black arrow: fibroblasts; E: epithelial tissue; K: keratin layer; GT: granulation tissue; B: capillaries with blood cells.

After 14 days, the wound samples were harvested and sectioned for histopathological analysis. Several important parameters for the wound healing process, such as inflammatory cell infiltration, re-epithelization, keratinization, granulation tissue, revascularization and remodeling [38], were observed from H&E stained slides (Figure 7). At 14 days after surgery, a large amount of inflammatory cells (indicated by red arrows) was still observed in the ColME, GelMA and control groups, indicative of prolonged inflammation caused by the infection, while a great decrease in infiltration of inflammatory cells was observed in the ColME-NIS and ColME-LEV groups, along with increasing fibroblasts (spindle-shaped, indicated by black arrows), that are responsible for collagen synthesis and deposition. Re-epithelization is a pivotal process in wound healing. In all groups, except the control group, the development of new epithelial tissue (denoted as E) was observed, leading to complete coverage of the wound area. Conversely, only partial re-epithelization was observed in the control group, indicating a less advanced stage of wound closure in comparison with the others. Additionally, not only was re-epithelization observed in all treatment groups, but also the occurrence of keratinization. Keratinization was characterized by the presence of either loosely adherent or exfoliated layers of keratin (denoted as K), as well as the development of a thickened parakeratotic stratum corneum layer on the outermost surface above the epidermis. The presence of granulation tissue (denoted as GT) was observed in all groups, however, the ColME, GelMA and control groups showed disorganized granulation tissue filling the incisional space, suggesting potential challenges or delay in wound healing due to infection. On the contrary, the wound treated with ColME-LEV and ColME-NIS hydrogels demonstrated more organized granulation tissue, indicating a well-coordinated healing process. Notably, these groups also exhibited the presence of capillaries with blood cells (denoted as B) and hair follicles, indicative of remodeling.

**Figure 8:**
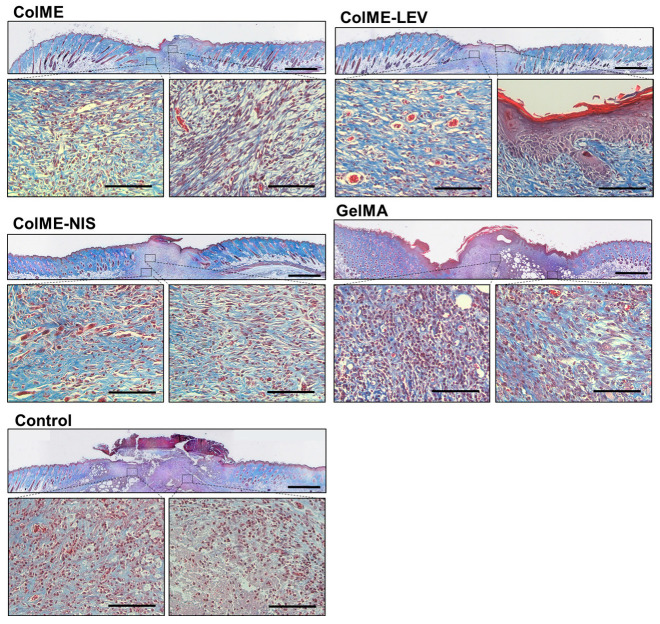
Representative Masson’s trichrome staining images on day 14. Scale bars in the low-magnification images, 1 mm; scale bars in the high-magnification images, 100 μm.

The deposition of collagen at the wound sites, which plays a vital role in the ultimate wound repair, was observed in the sections stained with Masson’s trichrome (Figure 8). Less collagen deposition was found in the ColME, GelMA and control groups, compared to that in the ColME-LEV and ColME-NIS. In summary, histological analysis revealed that antimicrobial hydrogel wound dressings exhibited reduced inflammation, improved re-epithelization, promoted granulation tissue formation, and enhanced collagen deposition. These findings highlight their potential for successful healing of infected wounds.

## 4. Conclusions

In this investigation, we have created an antimicrobial collagen hydrogel employing photo-crosslinking techniques. The hydrogel precursor, ColME, was combined with antimicrobial agents such as levofloxacin and nisin, forming hydrogel upon exposure to UV irradiation. These hydrogels have demonstrated both biocompatibility and efficacy in eradicating bacteria, both in vitro and in vivo. Specifically, hydrogels containing levofloxacin or nisin have effectively thwarted the development of abscesses and biofilms, while also promoting the healing of infected wounds in a mouse skin model. 

In conclusion, these results highlight the promising potential of antimicrobial hydrogels for advancing wound management, showcasing their ability to combat infections and support the healing process effectively. Despite considerable progress in this research, there are still challenges to be addressed in the application of these antimicrobial collagen hydrogels. Future research will focus on optimizing UV crosslinking for larger wounds, exploring alternative antimicrobial agents for broader efficacy, and assessing cytocompatibility with keratinocytes and capillary endothelial cells to enhance the comprehensive understanding and application of antimicrobial collagen hydrogels.

## Data Availability

Data is contained within the article.

## Supplementary Materials

The following supporting information can be downloaded at: https://www.mdpi.com/article/10.3390/polym15244701/s1, Figure S1: The physical appearance of ColME hydrogel. Figure S2: FTIR spectra of ColME, nisin, levofloxacin, ColME-NIS and ColME-LEV. Figure S3: A. Photographs of wounds with or without hydrogel treatment on 0, 3, 7, 10 and 14 post wound creation. B. Wound area evaluation for untreated or treated groups. *n* = 4. Error bars represents standard deviation.
