# Supplementary material for: Development of an In Situ Photo-Crosslinking Antimicrobial Collagen Hydrogel for the Treatment of Infected Wounds
Source: Polymers (Basel). 2023 Dec 13;15(24):4701. doi: 10.3390/polym15244701 (PMC10748037; doi:10.3390/polym15244701)
Supplement: Supplementary file 1 [file polymers-15-04701-s001.zip › polymers-2650095-supplementary.pdf]

Supporting Information

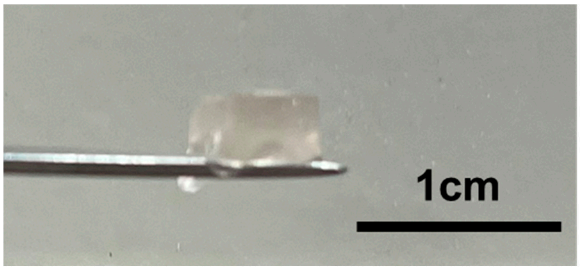

Figure S1. The physical appearance of ColME hydrogel.

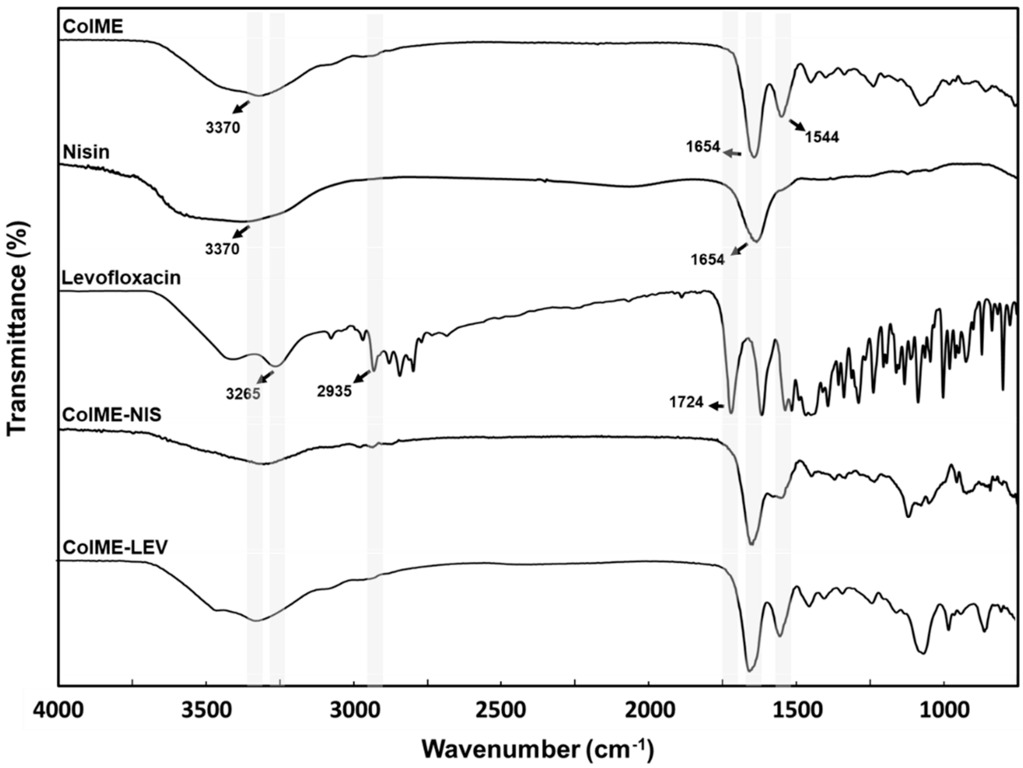

Figure S2. FTIR spectra of ColME, nisin, levofloxacin, NIS and LEV.

The ColME hydrogel was tested in a rat model of wound healing. The rats were divided into 3 groups: control (no treatment, n = 4), ColME hydrogel (n = 5) and GelMA hydrogel (*n* = 5). The procedure was described in the Materials and Methods. The results indicate that ColME hydrogel improved wound healing.

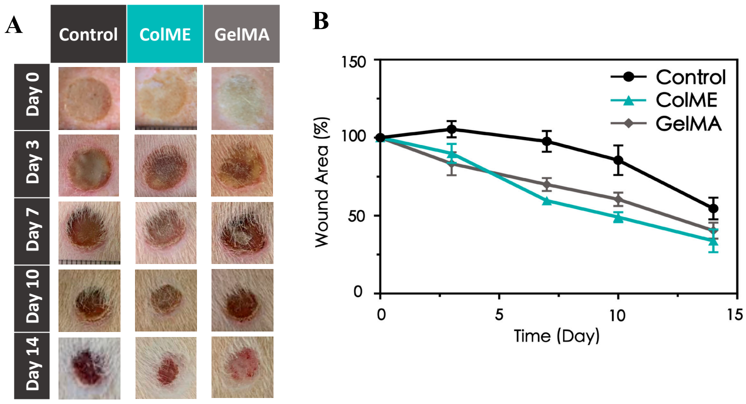

Figure S3. A. Photographs of wounds with or without hydrogel treatment on 0, 3, 7, 10 and 14 post wound creation. B. Wound area evaluation for untreated or treated groups. *n* = 4. Error bars represents a standard deviation.
